# The Upside of Failure: How Regional Student Groups Learn from Their Mistakes

**DOI:** 10.1371/journal.pcbi.1003768

**Published:** 2014-08-07

**Authors:** Tarun Mishra, R. Gonzalo Parra, Thomas Abeel

**Affiliations:** 1 Bioinformatics Division, School of Bio Sciences and Technology, VIT University, Tamil Nadu, India; 2 Protein Physiology Laboratory, Facultad de Ciencias Exactas y Naturales, Universidad de Buenos Aires, Buenos Aires, Argentina; 3 Broad Institute of MIT and Harvard, Cambridge, Massachusetts, United States of America; 4 VIB Department of Plant Systems Biology, Ghent University, Ghent, Belgium

## Abstract

Success is the result of planning, hard work, determination, foresight, and a little bit of luck. Unfortunately, nobody has thought to pave the road to success. Although failure can be discouraging and time-consuming, it presents incredible learning opportunities—the biggest difference between those who succeed and those who abandon their projects lies in their response to adversity. This article reviews events undertaken by the Regional Student Groups (RSGs) in India and Argentina, the problems they encountered, and what can be learned from them. RSG-India attempted to organize an online scientific meeting (also known as a virtual conference) with geographically dispersed stakeholders, a totally new concept for them. RSG-Argentina tackled the challenge of organizing a two-day symposium, their first event ever. Some of the complications they faced were easy to fix, others led to the cancellation of activities, and all of them resulted in valuable lessons. The main goal of this article is to highlight, through their experiences, the universal importance of a healthy panel of contingency plans.

## Introduction

Winston Churchill once said “Success is stumbling from failure to failure with no loss of enthusiasm.” Failure is a crucial opportunity to learn and develop. Reports from the scientific community generally focus on success and gloss over setbacks—negative results are rarely published, and it is even less common to read about failed paths of investigation en route to the final result. This façade of uninterrupted success suppresses the harsh reality that failure is a common—and ultimately useful—occurrence. This article presents a brutally honest and hopefully enlightening account of the problems that two student groups ran into while organizing various scientific events, as well as the valuable lessons learned from the pitiless experience of failure.

Regional Student Groups (RSGs) of the International Society for Computational Biology (ISCB) Student Council have been instrumental in organizing student-led activities aimed at developing the careers of young scientists in the field of bioinformatics. For many of the protagonists in these anecdotes, it was the first time that they took on the challenges of planning events on this scale. RSG-India and RSG-Argentina demonstrate from their first-hand experiences that ambition, hard work, and willpower do not always lead to success—a sobering lesson. You also need competence, foresight, and flexibility, and these qualities are only attainable through experience.

What follows is a forthright account of the doomed projects undertaken by RSG-India and RSG-Argentina, documented in the hope and expectation that others may sidestep these pitfalls and discover novel and exciting calamities with which to contend.

## RSG-India's Plans to Organize a Virtual Conference

### Project introduction

RSG-India is one of the largest operating groups and has been organizing a variety of successful activities since 2006. Inspired by two previous virtual conferences in Africa and India, they decided to join forces with groups from Pakistan and Australia to replicate these successes.

A virtual conference is an online meeting where presentations are done with video conferencing technology. Attendees can watch the talks through the website and ask questions through a chat-system. What is particularly appealing about this type of event is that people can attend without the need for a costly venue and travel expenses.

After several months of hard work, the virtual meeting of RSG-India was abandoned. Gaping holes in the planning emerged when the event started to take shape.

### Problems

The virtual conference project was hobbled from the beginning by a poorly defined goal. The organizing team had heard about the successes of other virtual conferences but did not further look into these events or their logistics, so they did not have a blueprint for how to organize such a meeting. They had, in other words, a half-baked idea. When outside participants and supporters were approached with the idea, they sensed its prematurity and politely, but speedily, ran away.

The second problem was the lack of internal organization. The team kept expanding, but lacked direction and leadership. Even after several months of planning, there were no committees and there was no clear division of tasks. This meant that team members had no personal responsibilities and as a result, did not get invested in the project. This was particularly problematic for the fundraising effort since nobody was in charge of coming up with a budget or contacting sponsors.

The final problem was the lack of proper tools and the breakdown of communications as a result. When executing a project with a large team, it is crucial to have centralized and organized means of communication. Online project management tools are a necessity to work with teams that span several time zones and have varying levels of commitment to the project. Using email as a primary means of communication led to confusion and resulted in people not being able to keep track of discussions through neverending email threads.

These problems combined resulted in the inevitable failure of this event, and the virtual conference never happened.

## RSG-Argentina's First National Student Meeting

### Project introduction

RSG-Argentina is a newly minted group with more ambition than experience. In 2012, they endeavored to organize their first national student meeting.

They aimed high. The first national meeting was going to be a two-day gathering with workshops, a round table discussion about the lack of private initiatives in the field of computational biology, and a student symposium where undergraduate students could present their work. They also planned to sell merchandise to raise money for future activities. Everything seemed straightforward when they were writing the proposal, but then reality hit them hard.

### Problems

RSG-Argentina set out to organize three two-day courses. Because many people wanted to attend the courses, the program was changed to accommodate six one-day courses instead, and they would be organized in the largest rooms available in the university.

The first disappointment came when it was clear that a noncommittal expression of interest does not guarantee actual attendance. Although there was a formal registration system, there was no sign-up fee, so there was little incentive for people to show up. As a result, the turnout was far lower than anticipated, much of the extra resources went unused, and some of the teachers presented their courses in near-empty halls.

During the different courses, participants would get access to computers provided by the university. Unfortunately, the day before the workshops it was discovered that the available hardware was not compatible with the software some of the instructors planned on using. It was too late to change the venue, and it is only thanks to the hard work of the university's system managers that the computers were sufficiently usable before the kick-off.

A third error in judgment led to the cancellation of the round table discussion with the invited speakers. It was assumed that the lecturers would be available throughout the day and not just for their lecture time. It turned out that some of them did have other plans and they could not participate in the afternoon panel discussion.

When it looked like things could not get any worse, they did. The organizers had to cancel the symposium because of lack of submissions. The advertising campaign did not start until a few weeks before the event, and it was too little, too late. There would have been plenty of attendees due to the workshop component, but without students presenting their science there was no prospect of a successful discussion.

On top of all that, the organizers had some bad luck with one of their contractors. Attendees could pre-order a customized T-shirt which they would receive when coming to the workshop. Unfortunately, the T-shirt sizes mentioned on the website were inaccurate. As a result, many people no longer wanted their ill-fitting T-shirt and demanded a refund, leaving the organizers with a stock of unsold T-shirts and a bill from the factory.

Finally, the Argentinian government suddenly instituted a new national holiday at the same time as the event that shut most of the country down, including most of the transport facilities needed by the workshop participants.

In spite of the seemingly endless torrent of torments, RSG-Argentina still felt like they came out ahead. While the four workshops they ended up offering were a far cry from their initial ambitious plan, they learned to work as a team. The next time they organize an event they will not be beginners anymore, and they surely will not be making any of these mistakes again.

## Lessons Learned

While this article discussed two specific events, the lessons that the organizers took away from their failures are broadly applicable.

### Due diligence

Regardless of the type of event, it is advisable to perform due diligence and research the state of the art. It is critical to define the scope of the event and its target audience. Talking to organizers of similar events is a great way to start. Generally this can be done by a single person who will be coordinating the event. It is crucial to have a blueprint before involving a large team or setting up international collaborations.

### Leadership and ownership

Teams with clear leadership and members who feel like part of the team have the best chance to survive an unexpected crisis. A random group of volunteers does not become a smoothly running event-organizing machine overnight. It takes some time for leaders to emerge, for team members to get involved, to get to know each other, and to take ownership of the activities and responsibilities that fit them the best. In this sense less is always more; start working with a small group on small events and take it from there. In particular for new groups, it is probably a good idea to learn how to walk before trying to run. It is better to split a large project into smaller initiatives to assure the success of each of these ventures—or to limit the damage in case of failure—before embarking on large, integrated events. This will allow the group to gain solid experience in specific tasks, improve their management skills, and design efficient strategies to face bigger projects in the future.

### Time management

Time is the single most valuable asset the group may or may not have when trouble appears. To ensure that there is time to address problems it is important to have the right team and a timeline to guide milestones in the project. Milestones ensure that critical tasks can be completed in a timely manner; e.g., preparing a budget goes before contacting the sponsors. The easiest way to build a timeline is to work backwards from the date of the event. Make sure to include generous padding in the timeline, which will be the time for fixing problems, extending deadlines, and chasing reviewers. It is also important to keep in mind that RSGs are entirely run by volunteers. Since there is no motivation in the form of a paycheck, it is extra important to have people on the team who have personal reasons to make the event a success. Nonetheless, life and paid work occasionally get in the way of volunteer work, and it is a good idea to give people some leeway and to build sufficient flexibility into the plan to deal with anticipated delays.

While it is important to stay hopeful and enthusiastic, it is equally important to know when to give up. When you have nothing to show for months of discussions, it is time to either dramatically change your approach or admit defeat.

### Communication

Last but not least is the importance of communication. Communication should be clear and timely. Active listening by summarizing the main discussion points helps to ensure mutual understanding. The primary goal is to avoid confusion and assumptions. Many of the mistakes that were made in these two examples could have been avoided with better communication, both between team members and with outsiders such as speakers and participants.

Timely communication and advertisement ensure that deadlines are met, that the target audience knows when abstracts are supposed to be submitted, and even more important, when and where the meeting will take place.

## Conclusion

While the two events we discussed either failed to materialize or were far from the original plan, they did provide excellent learning opportunities for both RSG-India and RSG-Argentina.

Planning is essential to organize a successful event. Make sure to research what others have done, how they have done it, and what the outcome was. However, be ready to be flexible when dealing with adversity. Very few plans survive the real world in their original form, and it will be necessary to adapt to deal with problems. In our experience, the success of an event depends more on how the organizers can deal with the problems than on the quality of the initial plan. Thinking about all potential problems is what planning is all about. Even then, it will be impossible to prepare for every eventuality.

Event organizing is not all doom and gloom. Most events do run successfully, and the feeling of overcoming all those challenges along the way is exceptionally gratifying. Sitting together with the team over drinks at the end of it all, reminiscing over how far you have come and how much you have learned, makes it all worth it. The friendships forged when conquering difficulties will last a lifetime.

**About the Authors**

The authors have worked on many aspects of the ISCB Student Council and the Regional Student Group program.

**Tarun Mishra** was president of RSG-India (2010–2012).

**R. Gonzalo Parra** was co-founder and president of RSG-Argentina (2012–2013).

**Thomas Abeel** was co-founder, president (2009), and secretary (2010–2011) of RSG-Europe and served as student representative to the ISCB Board of Directors from January 2011 to January 2013.

